# Dopamine-Sensitive Anterior Cingulate Cortical Glucose-Monitoring Neurons as Potential Therapeutic Targets for Gustatory and Other Behavior Alterations

**DOI:** 10.3390/biomedicines12122803

**Published:** 2024-12-10

**Authors:** Edina Hormay, Bettina László, István Szabó, Kitti Mintál, Beáta Berta, Tamás Ollmann, László Péczely, Bernadett Nagy, Attila Tóth, Kristóf László, László Lénárd, Zoltán Karádi

**Affiliations:** 1 Institute of Physiology, Medical School, University of Pécs, H-7624 Pécs, Hungary; bettina.csetenyi@aok.pte.hu (B.L.); beata.berta@aok.pte.hu (B.B.); tamas.ollmann@aok.pte.hu (T.O.); zoltan.karadi@aok.pte.hu (Z.K.); 2 Medical and Engineering Multidisciplinary Cellular Bioimpedance Research Group, Szentágothai Research Centre, University of Pécs, H-7624 Pécs, Hungary

**Keywords:** anterior cingulate cortex, glucose-monitoring neuron, dopamine, streptozotocin, behavior

## Abstract

**Background**: The anterior cingulate cortex (ACC) is known for its involvement in various regulatory functions, including in the central control of feeding. Activation of local elements of the central glucose-monitoring (GM) neuronal network appears to be indispensable in these regulatory processes. Destruction of these type 2 glucose transporter protein (GLUT2)-equipped chemosensory cells results in multiple feeding-associated functional alterations. **Methods**: In order to examine this complex symptomatology, (1) dopamine sensitivity was studied in laboratory rats by means of the single-neuron-recording multibarreled microelectrophoretic technique, and (2) after local bilateral microinjection of the selective type 2 glucose transporter proteindemolishing streptozotocin (STZ), open-field, elevated plus maze, two-bottle and taste reactivity tests were performed. **Results**: A high proportion of the anterior cingulate cortical neurons changed their firing rate in response to microelectrophoretic administration of D-glucose, thus verifying them as local elements of the central glucose-monitoring network. Approximately 20% of the recorded cells displayed activity changes in response to microelectrophoretic application of dopamine, and almost 50% of the glucose-monitoring units here proved to be dopamine-sensitive. Moreover, taste stimulation experiments revealed even higher (80%) gustatory sensitivity dominance of these chemosensory cells. The anterior cingulate cortical STZ microinjections resulted in extensive behavioral and taste-associated functional deficits. **Conclusions**: The present findings provided evidence for the selective loss of glucose-monitoring neurons in the anterior cingulate cortex leading to motivated behavioral and gustatory alterations. This complex dataset also underlines the varied significance of the type 2 glucose transporter protein-equipped, dopamine-sensitive glucose-monitoring neurons as potential therapeutic targets. These units appear to be indispensable in adaptive control mechanisms of the homeostatic–motivational–emotional–cognitive balance for the overall well-being of the organism.

## 1. Introduction

The rapidly increasing incidence of various feeding–metabolic diseases (diabetes mellitus, obesity, metabolic syndrome, etc.) poses an extreme burden on the healthcare systems of modern societies [1,2,3]. In such situations, any scientific breakthrough in the control of feeding and metabolism would raise new hopes of getting closer to developing more effective prevention and better therapeutic approaches. Related studies in our field so far have focused mainly on peripheral pathological mechanisms in the background of the homeostatic disturbances usually characterizing these disorders [4,5,6,7], although recently, especially after re-discovering the classic work of Mayer from the 1950s [8,9,10], the causal significance of central regulatory abnormalities is also becoming more frequently emphasized [8,9,11,12,13]. At this point, one of the most intriguing problems with these kinds of investigations is the lack of research addressing the issue of how, and by which brain structures, the necessary cooperation among regulatory mechanisms of the homeostatic and motivational–behavioral control processes of feeding and metabolism takes place in the healthy organism. The present investigations, therefore, were designed to combine both of the above aspects by placing the currently poorly studied anterior cingulate cortex and its dopamine modulation in the center of this research.

Glucose, as one of our most fundamental energy sources, plays a distinguished role not only in basic metabolic processes but also in the control of the abovementioned regulatory mechanisms of food and fluid intake behavior [10,14], by utilizing the activation of specific receptors of certain internal organs in the periphery and those within the central nervous system as well [15,16,17]. Its distinguished role is exerted in these control processes by cells that are responsible for continuous detection of glucose concentration in the blood as well as in the local interstitial space of certain brain areas [8,9,10]. In the central nervous system, these specific, pancreatic β-cell-like chemosensory neural cells [18], the so-called glucose-responsive (or glucose-excited and -inhibited) or, in a more real function-elucidating nomenclature, glucose-monitoring (GM), neurons [10,11,19] change their firing rate along with changes in the blood glucose level or in response to microelectrophysiological as well as local intracerebral or systemic administration of D-glucose [10,15,16,17]. As their characteristic functional attribute, a great majority of these forebrain chemosensory (GM) neurons express the type 2 glucose transporter protein (GLUT2)—the principal glucose transporter here—to take glucose intracellularly [20,21,22,23].

Taking into consideration the aims of this study, it appears to be unavoidable to search for such GM units in our target limbic structure also, in the anterior cingulate cortex. A virtual problem that one should solve in planning the appropriate experimental protocol is that the ACC has been known to play important roles in behavioral–cognitive regulation [24,25,26], whereas the glucose-monitoring neurons in the limbic forebrain are also broadly discussed concerning their involvement in homeostatic–metabolic functions [11,13,27]. Recent studies, however, elucidated the homeostatic relevance of the ACC as well, showing alterations in local blood flow and the glucose metabolism of the anterior cingulate cortical area in the case of some widely studied neuropsychological diseases, such as major depressive disorder, schizophrenia, autism, and post-traumatic stress disorder [28,29,30,31].

Given the extraordinarily rich morphological and functional inter-relationships of the anterior cingulate cortex (with special emphasis on contributions of its glucose-monitoring neurons and their dopaminergic neuromodulation) [24,32,33], it is now reasonable to suppose that the local chemosensory units, along with their dopamine neurotransmission, are very much intimately involved [24,25,26] in the above complex and in reward-associated regulatory functions. It is logical to investigate how the glucose-monitoring neurons in this area play a role in the regulation of behavioral functions [26,33,34].

The catecholamine dopamine is one of the most thoroughly examined neurotransmitters of the mammalian brain, where it participates in the control of a great variety of functions including the regulation of reward, diverse surveillance of motivational and learning processes, emotion, cognition, positive reinforcement and, among others, the integration of relevant factors in the decision-making process of setting a given behavior output, in relation to feeding as well [11,19,35,36,37,38,39]. The major brain sites to host dopamine-producing (dopaminergic) neurons are the ventral tegmental area, the substantia nigra pars compacta, and the retrorubral area, the A10-A9-A8 cell continuum. Another minor DA neuron line was identified in the arcuate nucleus of the hypothalamus. The axons of these neurons have already been found in various areas of the brain, with direct projections into numerous cortical and subcortical limbic structures, including the prefrontal cortex, nucleus accumbens, basolateral and central amygdala, ventral pallidum, hippocampus, globus pallidus, substantia nigra, caudate nucleus and the putamen [37,40,41]. Several of the above dopaminergic structures have a clear overlap with elements of the forebrain GM neuronal network [17,22,38,42,43,44]. Furthermore, as an obvious sign of the strong functional inter-relationship of the two systems, catecholamines, especially dopamine, have already been shown to significantly modulate forebrain GM neurons [10,11,19,22,42,44].

As far as the relevance of the gustatory part of this study is concerned, the cingulate cortex (CC) has already been shown to be involved in high-level taste information processing. First, Rolls and his co-workers provided evidence for the existence of gustatory-stimuli-driven anterior cingulate cortical neural cells [45] in the primate, monkey brain, and later, in our microelectrophysiological pilot experiments more than a decade ago, we also recorded taste-responsive neurons in the rodent ACC [46,47]. The participation of this cortical area in high-level control of gustation was further underlined by results of functional MRI investigations that provided evidence for pleasant/ingestive and unpleasant/aversive gustatory-stimulation-induced differential activation of the CC even in the human brain [48,49]. These findings demonstrated that the cingulate cortex plays a distinguished role in centrally organized broad gustatory functions; thus, it appeared to be reasonable to design the present experiments in the way we did, that is, to set them to examine relatively simple and also more complex aspects of the central taste information processing and the control of related behaviors.

The implementation of general behavioral paradigms in the present study is well explained by a great amount of experimental and clinical data, indicating that the ACC is a functionally diverse area which, nevertheless, is known characteristically for its involvement in motivational control and in the regulation of original emotional and cognitive processes, interconnecting the emotional and cognitive components of the behavior. The anterior cingulate cortex participates in the organization of motivated attention and attention allocation, as well as in the evaluation of reward value and reward-guided action selection [26,50,51,52,53,54]. It is also worth noting that the deficits of these and related emotional–cognitive control processes are considered to also, as a matter of fact, play an important role in the symptomatology of recently broadly and intensively studied disorders, such as schizophrenia [55], depression [30,56] or post-traumatic stress disorder [32].

Taking all of the above into consideration, the present study was designed to investigate the broadly manifesting, differential and significant functional characteristics of the local chemosensory neurons in two major lines of experiments. On the one hand, the most authentic neurochemical attribute of ACC units, the neuronal dopamine sensitivity, has been examined in adult male Wistar laboratory rats by means of the extracellular single-neuron-recording multibarreled microelectrophoretic technique. On the other hand, to characterize the system-level or behavioral-type functional deficits that follow the selective destruction and consequent loss of the GM cells in the ACC, open-field, elevated plus maze, two-bottle and taste reactivity tests were performed before and after local bilateral microinjection of the selective GLUT2-demolishing streptozotocin (STZ). STZ, with a nearly analogous molecular structure, strikingly resembling that of the D-glucose, is known to enter intracellularly via ‘ordinary’ activation of GLUT2 and then cause the death of these chemosensory cells by inducing an inter-related self-triggering series of oxidative stress mechanisms and DNA alkylation [21,23,57,58,59].

## 2. Materials and Methods

### 2.1. Experimental Design, Basic Conditions

**Figure 1:**
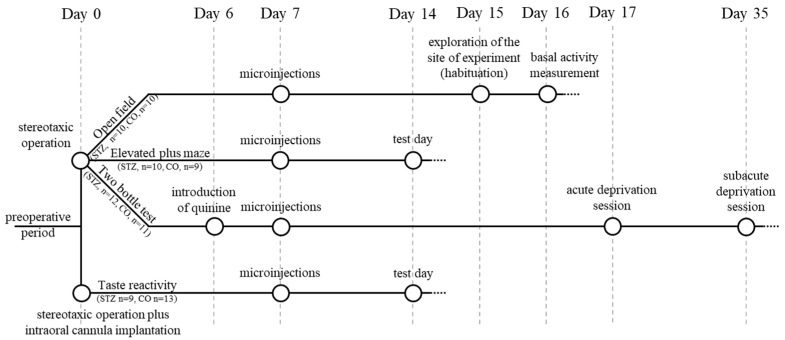
Comprehensive scheme of the behavioral experimental design.

Experiments of the present study started, in total, with 75 male Wistar laboratory rats, with an average of 250–300 g body weight at the beginning of the examinations. The study itself was divided into two examination lines: 24 animals were used for microelectrophysiology, and 51 for behavioral experiments. During the behavioral tests, animals, in a random order and distribution determined by average body weight, were assigned to two major—streptozotocin-treated (STZ) and control (CO)—groups. In detail, as seen in Figure 1, 23 animals (STZ, n = 12, control, n = 11) were used for the open-field, the elevated plus maze, and the two-bottle tests, and 22 rats for the taste reactivity tests (STZ, n = 9, control, n = 13). Due to various reasons, such as procedural mistakes or extreme complications, failure of anesthesia during surgery (n = 2) or after histological verification of misplacement of the microinjection cannula (n = 4), the results of six subjects were excluded from the final 45-animal data pool of this study.

The animals were kept in a light-, temperature- and humidity-controlled room (12:12 h light–dark cycles with light on at 7:00 a.m.; 21 ± 2 °C standard temperature; constant humidity at 55–60%), and cared for in accordance with institutional, national and international regulations (BA02/2000-8/2012, BA02/2000-55/2017, BAI/35/55-177/2017 University of Pécs, Medical School; Hungarian Government Decree, 40/2013. (II. 14.); NIH Guidelines, 1997; European Community Council Directive 86/609/EEC 1986, 2006; European Directive 2010/63/EU of the European Parliament).

### 2.2. Extracellular Single-Neuron Activity Recording, Administration of Neurochemicals, Gustatory Stimulus Delivery

Stereotaxic-surgery-assisted microelectrophysiological experiments were performed under long-term anesthesia in adult male Wistar laboratory rats (n = 24). The anesthesia was implemented by intraperitoneal injection of urethane (26%, 0.6 mL/100 g body weight; Sigma, Budapest, Hungary; Steinheim, Germany). During the stereotaxic operation, a tungsten wire glass multibarrel microelectrode was led into the ACC by means of a hydraulic microdrive (Narishige MO-10, Japan). The stereotaxic coordinates according to the stereotaxic rat brain atlas of Paxinos [60] were as follows: anteroposterior (AP), Bregma + 1.6–3.7 mm; mediolateral (ML) 0.3–1.6 mm; vertical (V) 0.9–2.9 mm from the brain surface.

**Figure 2:**
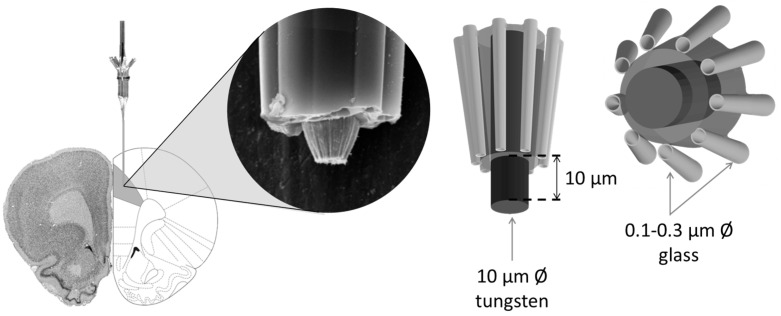
Experimental arrangement of the multibarreled extracellular single-neuron activity recording and the structural–mechanical design of the electrode.

The neuronal activity was recorded via the central barrel of the microelectrode (tungsten wire with tip of diameter 10 µm; impedance 5–8 MΩ at 50 Hz). This central barrel was surrounded by nine small glass capillaries (with holes of diameter 0.1–0.3 µm at the tip), from which neurochemicals were ejected into the surrounding extracellular space of the examined neuron (Figure 2). One of the nine capillaries worked as a current balancing channel (NaCl 0.9%), and another one marked the recording position of the microelectrode tip (methylene blue solution, 4%). The neurochemicals were applied electrophoretically, and each remaining barrel was filled with one of the following solutions: D-glucose (0.5 M, pH 7.0), dopamine hydrochloride (0.5 M, pH 6), and monosodium L-glutamate (0.5 M, pH 7–8; to test the electrode tip’s vicinity to the recorded neuron). The neurochemicals were ejected from their respective barrels by constant currents of appropriate polarity (ejection current intensities: ± 30, 60, 90 nA; Neurophore BH2, Medical Systems, Great Neck, NY, USA). The recorded action potentials were amplified, filtered (Supertech Ltd., Soltvadkert, Hungary; Guben, Germany), and digitalized (CED 1401 mk II, Cambridge, UK). The neuronal signal was processed by the Spike 2 v6.16 software package (Cambridge Electronic Design Ltd., Cambridge, UK).

Only the well-isolated signals of spontaneously firing neurons were recorded for real-time and offline analyses, corresponding to the internationally recognized analysis criteria of our laboratory [43]. The baseline activity of a neuron was calculated from the discharge frequency of 4 to 7 characteristic, non-stimulation, resting periods with the duration of 10–30 s. Neuronal response (firing rate change) criteria to a certain “stimulus“ (neurochemical administration, taste stimulus delivery) are as follows: at least ±30% change from the mean baseline firing rate level; dose dependence; and replicability.

With respect to neuronal responsiveness, microelectroosmophoretic administration of D-glucose (0.5 M, pH 7) elucidated the existence of 3 types of neural cells in the ACC: one with excitatory, and another one with inhibitory response (the GR/GE and the GS/GI kinds of GM units, respectively). In the third group, the non-responding cells were identified as glucose-insensitive (GIS) units, using the glucose exclusively as an energy resource to maintain their metabolism.

In case of intraoral gustatory stimulation, a polyethylene tube (Hibiki 3, HIBIKI, Tokyo, Japan) was implanted into the mouth of the anesthetized rat [11,44,58], and the neuronal responsiveness was examined by intraoral infusion of various taste solutions of the five primary taste qualities and orange juice in two concentrations: sweet (sucrose; 0.1 M, 0.3 M); salty (NaCl; 0.1 M, 0.3 M); sour (HCl; 0.01 M, 0.03 M); bitter (QHCl; 0.00a1 M, 0.003 M); umami (MSG; 0.1 M, 0.3 M); and orange juice (complex taste; 10%, 25%). Stimulus sample volume was 0.5 mL; the stimulus delivery rate was 0.5 mL/min. Each taste stimulation was followed by rinses with distilled water and an air puff. The interstimulus time interval was set at one minute.

### 2.3. Behavioral Investigations (Operation; Intracerebral Microinjection; Behavioral Tests)

#### 2.3.1. Stereotaxic Surgery

During the stereotaxic surgeries, general anesthesia was administered by intraperitoneal injection of the 4:1 mixture of ketamine (Calypsol, 50 mg/mL; 3 mL/kg body weight; Richter Gedeon Rt., Budapest, Hungary) and diazepam (Seduxen, Richter Gedeon, Hungary, 5 mg/mL; 0.6 mL/kg body weight).

Stainless steel guide cannula (23 G) were placed bilaterally on the surface of the dura above the ACC according to the stereotaxic rat brain atlas of Paxinos [60] (AP: Bregma + 2.7 mm, ML: 0.9 mm, V: 0 mm) with a fine mechanical microdrive (MN-33 Narishige, Japan), and attached to the skull with dental acrylic (Duracryl, Ostrava, Czech Republic) as well as stainless steel anchoring screws. Animals were kept individually after the operation, even during the acute, one-week-long postoperative recovery period.

#### 2.3.2. Microinjection

After 12 h of food withdrawal, thin (30 G) stainless steel delivery cannula were penetrated through the guide cannula and positioned bilaterally above the required site (Figure 6, V: 1.6 mm from the brain surface) to administer 0.75 μL STZ (Sigma (Budapest, Hungary; Steinheim, Germany) S-0130, 10 μg/μL; 7.5 μg STZ dissolved in sterile physiological saline) or sterile physiological saline directly into the ACC in the alert, hand-held rats. The microinjection cannula were connected through a polyethylene tube (HIBIKI 3, Hibiki Co., Tokyo, Japan) to a 25 μL-capacity Hamilton syringe fixed in a microinfusion pump (Cole-Palmer EW-74900 Multichannel Syringe Pumps, Cole-Palmer Instrument Company, Vernon Hills, IL, USA), which was set to one-minute-long administration time duration. The delivery cannula, however, were temporarily kept in place even after the administration finished and were removed only after one more minute to allow the solution to fully diffuse into the target area [61], as well as to prevent the backflow of a non-negligible portion of the original volume after premature removal of the cannula. Behavioral experiments were implemented and started one week after the anterior cingulate cortical STZ or vehicle (control) microinjection, except in the case of those tests where an additional conditioning step was needed (Figure 1).

#### 2.3.3. Behavioral Tests

##### Open-Field Test

**Figure 3:**
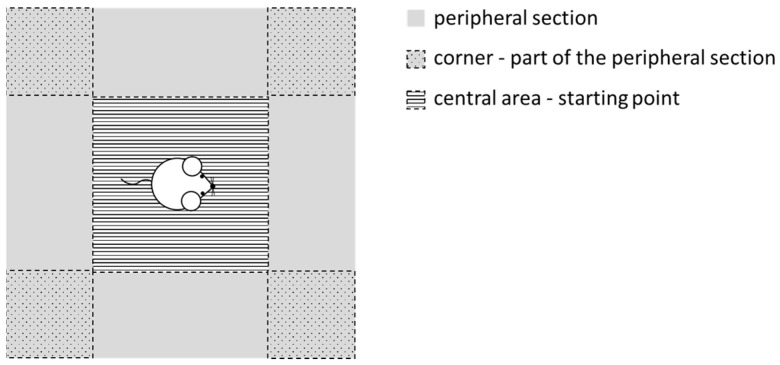
Experimental arrangement of the open-field test.

The rats were placed in a 50 × 50 × 50 cm dark grey wooden box, allowing them to move freely and enabling us to observe and record general locomotor activity, species-specific motor patterns (grooming, rearing, sniffing, etc.), as well as urination, defecation, and eventual vocalization of the animals. The floor area of the apparatus (assigned to a central area, four corners and a peripheral section corresponding to the edges of the box, including the four corners) was divided into 16 equal-size constituent parts utilized for the evaluation procedure of the habituation (introduction to the experimental area) and testing (basal activity measurement) sessions (Figure 3). The animals’ behavior was analyzed and evaluated by means of the Noldus Ethovision system and its Basic software (https://www.noldus.com/ethovision-xt, accessed on 24 November 2024) (Noldus Information Technology, B.V., Wageningen, The Netherlands). The results of the two (control and STZ-treated) groups were compared.

##### Elevated Plus Maze

**Figure 4:**
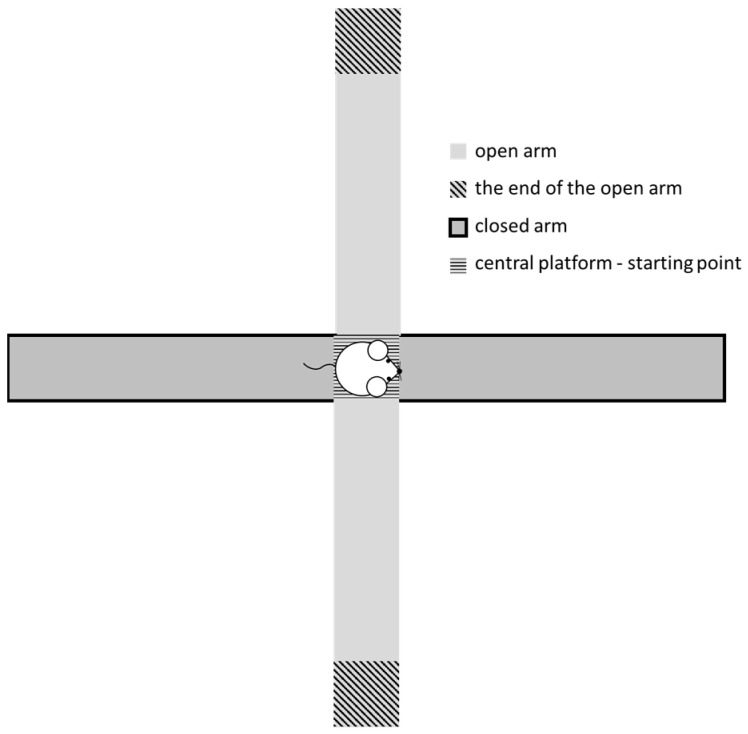
Experimental arrangement of the elevated plus maze test.

The elevated plus maze test is primarily used to evaluate anxiety levels of the individual, and we also duly employed it for analyzing the general behavior and movement of each animal after observing them in 5 min-long trials. In this study, we used a cross-shaped apparatus placed 1 m above the ground floor and consisting of two 50 × 10 cm dark grey wooden planks, one without walls—the so-called open arm—and the other with 40 cm high walls, serving as the so-called closed arm. The neighboring arms were fixed in an angle of 90°. The animals, while facing towards one of the closed arms, were placed on the central platform in the middle of the maze as a starting point (Figure 4). The time spent in the open- and closed-arm areas, as well as at the end of the open arm (end-arm) were measured and compared between the STZ-treated and the control groups. Data were stored and motion analysis was made by means of the EthoVision Basic software (Version number: 2.3.19).

##### Two-Bottle Test

The two-bottle test consisted of 3 parts: a free drinking period, and an acute as well as a subacute deprivation session.

Animals were kept individually in separate cages, and they could choose between two bottles during drinking, one containing tap water and the other filled with 0.03 mM quinine hydrochloride (QHCl) solution. In order to avoid place preference that would have necessarily developed if the bottles remained in place throughout the experiment, the position of the bottles was changed at random time intervals. The weight of each bottle was measured daily and recorded in order to assess the respective quinine and water consumptions. During the non-deprived session, the animals could drink from any one of the two bottles without any kind of restriction for 12 days. In the acute (1 week after the intracerebral microinjection) and the subacute (4 weeks after the microinjection) deprivation sessions, however, the bottles were removed from the cage, i.e., the animals were deprived of any fluids for 24 h prior to testing. At the start of such tests, both the quinine hydrochloride solution and the tap water were re-introduced, and the consumptions were measured and recorded, with attention to clearly differentiating these values in the first, second and third hour of drinking. The quinine and tap water consumptions of the STZ-treated and the control groups were compared and differentially evaluated during the non-deprived and the acute and the subacute deprivation sessions as well.

##### Taste Reactivity Test

Chronic intraoral taste cannula made of polyethylene tubes (HIBIKI 3, Hibiki, Japan), for later delivery of the various taste solutions, were first positioned in the oral cavity of rats allocated for the taste reactivity tests. The operation, just before starting the stereotaxic part of the surgery, was performed by first initiating anesthesia for the whole session. As described previously in detail (see at Section 2.3.1), the anesthesia was introduced by an intraperitoneal injection of 4:1 mixture of ketamine and diazepam. The gustatory cannula were placed anterolateral to the first maxillary molar and carefully led transbuccally, also subcutaneously, to emerge at the lateral aspect of the skull. Cannula were fixed in position by surgical stitches. Antiseptic solution (Betadine, EGIS, Budapest, Hungary) was applied locally when finishing all surgical interventions.

The animals, with their buccal cannula, were habituated to stay calmly inside a Plexiglas cylinder (30 cm diameter, 30 cm height), where they grew accustomed to the serial liquid infusions into their oral cavity at a constant flow rate (0.5 mL/min; Cole-Palmer microinfusion pump 789200C, Cole-Palmer Co., Ltd., Vernon Hills, IL, USA).

The taste reactivity test was performed 1 week after the intracerebral microinjection sessions. According to the method routinely used in our laboratory [62], the rats received two different (a lower and a higher) concentrations of taste solutions of the five primary taste qualities: sweet, sucrose (0.05 and 0.5 M); salty, NaCl (0.05 and 0.5 M); sour, HCl (0.03 and 0.3 M); bitter, QHCl (0.03 and 3.0 mM); and umami, MSG (0.05 and 0.5 M). After the infusion of 0.5 mL taste solution, the buccal cannula were cleaned by rinsing with distilled water and with one or two puffs of air.

**Figure 5:**
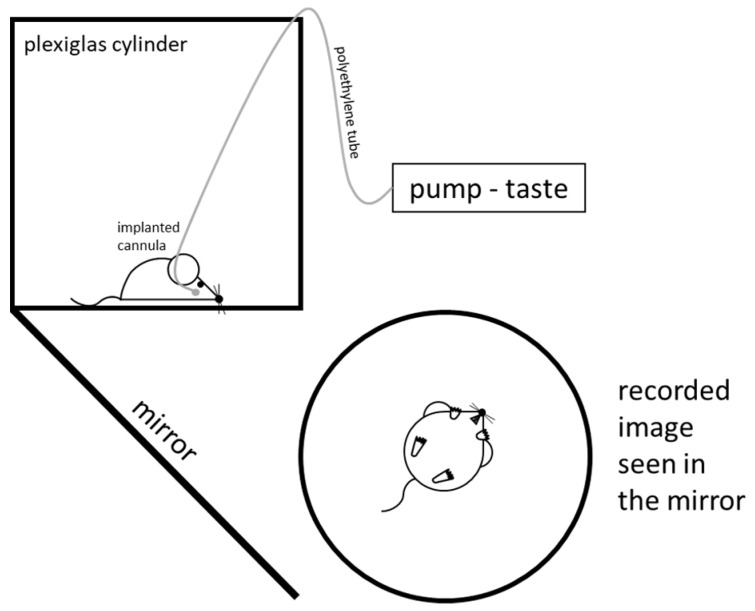
Experimental arrangement of the taste reactivity test.

In order to observe and evaluate the gustatory-stimuli-elicited reactions, primarily through the mouth and tongue movements of the experimental animals, a mirror was fixed at a 45° angle under the glass floor of the Plexiglas cylinder (Figure 5). The behavior of the rats was recorded with a video camera (Panasonic SDR-H85) (Panasonic, Osaka, Japan) and the recording was analyzed in a frame-by-frame manner (the frames corresponding to 25 ms time intervals). The observed species-specific mimicking and postural–locomotory patterns were differentiated into two distinct response types: rhythmic mouth movements, rhythmic tongue protrusions, lateral tongue protrusions and paw-licking were considered ingestive (accepting) reactions; gaping, head-shaking, paw-flailing, chin-rubbing and complex overexcited locomotory actions were considered as aversive (rejecting) reactions.

The modified protocol of Grill and Norgren [63,64] was used to perform a semi-quantitative analysis procedure in order to evaluate the taste reactivity behavior of the animals. Both aversive and ingestive patterns were judged and scored from level 0 to level 3, based on the occurrence, intensity, and duration of the given response, by at least three independent, experienced judges. Taste reactivity indices were calculated by dividing the average of the scores given by the referees by the maximum possible score (3). These taste reactivity indices were calculated for each gustatory stimulus.

The summed ingestive vs. aversive patterns were also evaluated; furthermore, responses were also differentiated as ones elicited by “pleasant” (mostly ingestive—0.05 M NaCl, 0.05 M MSG, 0.05 M sucrose, 0.5 M sucrose), “unpleasant” (mostly aversive—0.03 mM QHCl, 3 mM QHCl, 0.3 M HCl) or “hedonically complex” (both pleasant and unpleasant: a mixture containing tastants roughly eliciting equally ingestive and aversive responses—0.03 M HCl, 0.5 M NaCl, 0.5 M MSG) taste stimuli [65].

#### 2.3.4. Histology

**Figure 6:**
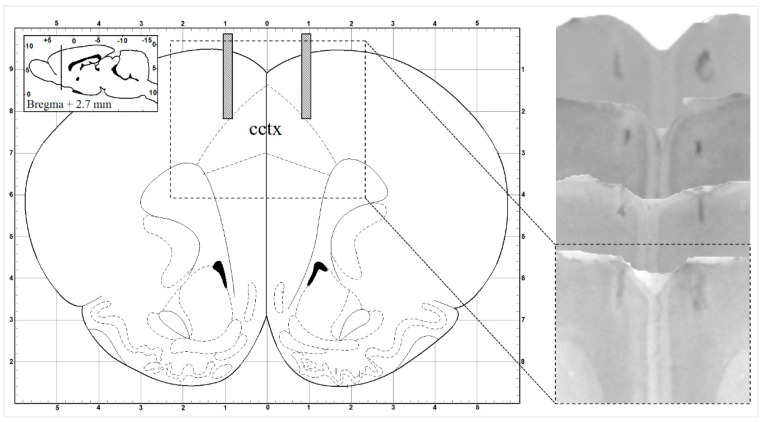
Localization of delivery cannula position: Scheme and rostrocaudally sequenced histological microphotograph examples of the bilateral anterior cingulate cortical microinjections [60].

To provide histological evidence for the successful microinjections, at the end of all experiments, the animals were euthanized by overdosed intraperitoneal injection of urethane (26%, fresh solution, 0.8 mL/100 g body weight; Sigma, Budapest, Hungary; Steinheim, Germany). After transcardial perfusion by physiological saline, followed by 10% formalin, the preserved brains were later cut into 40 µm sections and stained by Cresyl Violet (Nissl) [46] (Figure 6).

#### 2.3.5. Statistical Analysis of Data

To objectively support the well-studied interpretation of findings of the experiments, data analysis was performed by employing the SPSS for Windows software package (Version number: 22.0.0.0). The results are presented as means ± SEM, and the statistical significance of differences was set at levels *p* ≤ 0.05 (*) or beyond *p* ≤ 0.001 (**).

For statistical analysis of open-field and elevated plus maze tests, the Mann–Whitney U test was used. For analyzing the results of two-bottle and taste reactivity tests, the Kruskal–Wallis test and the Dunn test for post hoc comparisons were employed.

## 3. Results

### 3.1. Microelectrophysiology

During 39 recording sessions, the extracellular activity of 96 neurons was examined. A total of 15 (15.6%) of the tested 92 anterior cingulate cortical cells responded to microelectroosmophoretic administration of D-glucose, that is, acted as constituting local elements of the forebrain GM neuronal network. Out of these 15 cells, 11 neurons (73.3%) displayed an increase in firing rate and were identified as glucose-receptor (GR)- or glucose-excited (GE)-type cells. Four units (26.7%) were suppressed by the glucose application; thus, they were assigned as glucose-sensitive (GS)- or glucose-inhibited (GI)-type neurons. The microelectrophoretic administration of DA elicited differential responsiveness of the GM and GIS neurons in the ACC. As shown in Table 1, the former was significantly much more likely to change in activity in response to DA than the latter was (47% of the GM units vs. 13% of the GIS cells, *p* < 0.005, chi-square test).

**Figure 7:**
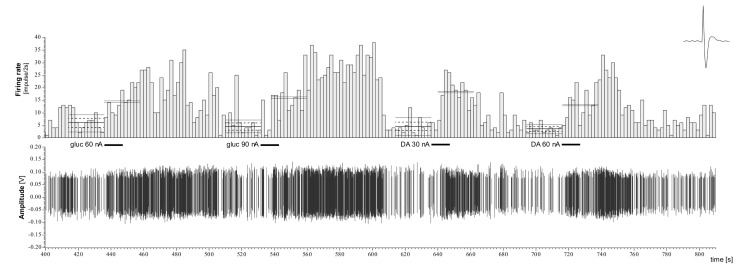
Dopamine excitation of a rat anterior cingulate cortical GR-type GM neuron. Thick horizontal bars: the duration of microelectrophoretic applications. Numbers: ejection current intensities. Abscissa: time in seconds [s]; ordinate: firing rate in spikes/2 s (upper trace) and electric potential in volt [V] (lower trace). Top right: characteristic extracellular action potential of the neuron. Mean baseline (prestimulus) activity of the neuron is marked by thin continuous solid lines, along with the ±30% response criterion (dashed lines), as well as the standard deviation of the firing rate (dotted lines). The average after-stimulation activity is marked by double solid lines.

Preliminary results of the gustatory stimulation experiments elucidated a robust (57%) taste sensitivity of the anterior cingulate cortical neurons tested. For all taste qualities, both the GIS and the GM units displayed high response rates (49% and 80%, respectively), but the analysis of the neurons’ responsiveness to individual gustatory qualities revealed a specific and very significant sensitivity of GM neurons to the bitter stimulus QHCl (*p* < 0.01, chi-square test) (Figure 7).

### 3.2. Open-Field Test

**Figure 8:**
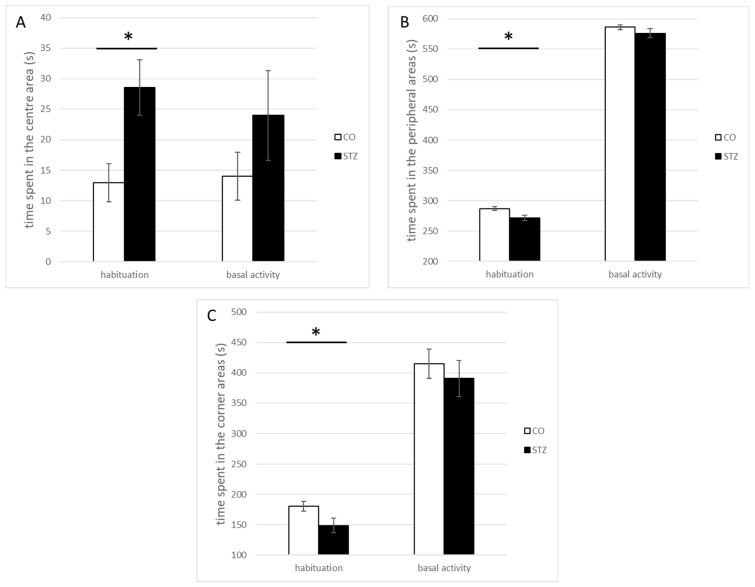
Demonstration of a characteristic behavioral shift in the ACC STZ-microinjected rats: In the habituation phase of the OF test (STZ group, more time spent in the (**A**) center, less time spent in the (**B**) periphery and at the (**C**) corners; U = 21, *p* < 0.05, in all three sub-regions of the apparatus. No considerable difference in the general locomotor activity of the two groups either in the habituation or in the baseline phases. (* means the statistical significance level of the differences at *p* ≤ 0.05).

The loss of GLUT2-equipped anterior cingulate cortical GM neurons resulted in remarkable changes in the locomotor activity of the rats. The STZ-treated animals (STZ, n = 10) spent significantly less time in the corners and the periphery, in contrast to what was found in the control group (control; n = 10): the neurotoxin-microinjected animals spent more time in the center of the arena, than the controls did. All changes were detected during the habituation phase of the test (center, STZ, 28.54 ± 4.85 s, control, 12.96 ± 3.11 s, U = 21, *p* < 0.05; periphery, STZ, 271.46 ± 4.58 s, control, 287.04 ± 3.11 s, U = 21; *p* < 0.05; corner, STZ, 148.66 ± 11.8 s, control, 180.57 ± 8.34 s, U = 21; *p* < 0.05) (Figure 8A–C). In the baseline activity measurements, at the same time, similar direction differences appeared to exist, but they did not reach the level of significance.

**Figure 9:**
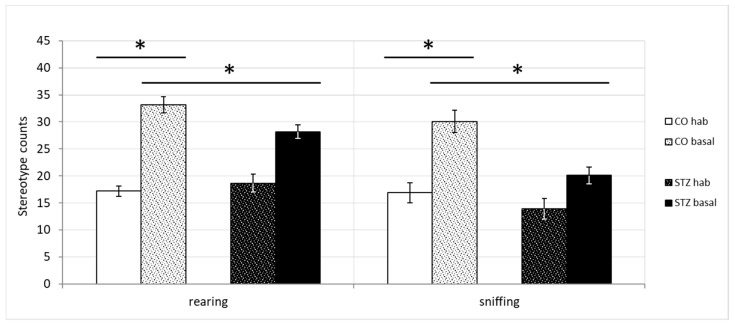
Significant differences in the sniffing and rearing activities: Although the performance of the control and STZ-treated animals was almost identical in the habituation phase, in the basal activity phase, the control group exerted much stronger rearing and sniffing activities compared to that of the STZ group. (* indicates the statistical significance level of the differences at *p* ≤ 0.05).

Significant differences appeared, however, between the baseline activity phase of control and both control and STZ habituation phases in the highly repetitive behaviors, namely in the rearing and sniffing stereotypes (rearing counts, control hab, 17.18 ± 2.49, control basal, 33.18 ± 3.27, STZ hab, 18.67 ± 2.6, STZ basal, 28.17 ± 4.87; Kruskal-Wallis, χ2(3) = 13.122, *p* < 0.05, control hab vs control basal, Dunn, *p* < 0.05, STZ hab vs control basal, Dunn, *p* < 0.05; sniffing counts, control hab, 16.91 ± 1.67, control basal, 30.09 ± 2.75, STZ hab, 13.92 ± 1.53, STZ basal, 20.08 ± 1.8; Kruskal–Wallis, χ2(3) = 17.582, *p* < 0.05, control hab vs. control basal, Dunn, *p* < 0.05, STZ hab vs. control basal, Dunn, *p* < 0.05) (Figure 9).

### 3.3. Elevated Plus Maze Test

**Figure 10:**
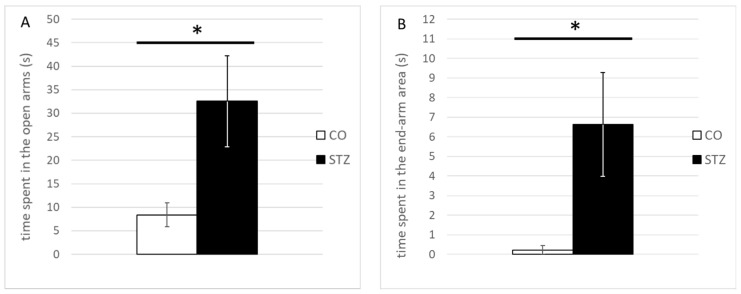
Significant group differences in the elevated plus maze test. Much longer time durations of the STZ-treated animals spent (**A**) within the open arms as well as (**B**) at their edge region in the elevated plus maze test. (* represents *p* ≤ 0.05).

The elevated plus maze test revealed unquestionable anxiolytic effects of the STZ microinjection into the ACC. The STZ-treated rats spent a significantly longer time in the open-arm area of the platform, also including the end stage (edge) of the open arm (open arms, total duration, STZ, 32.54 ± 9.66 s, control, 8.36 ± 2.54 s, U = 21; *p* < 0.05; open-arm edge, total duration, STZ, 6.62 ± 2.66 s, control, 0.22 ± 0.22 s, U = 25; *p* < 0.05) (Figure 10).

### 3.4. Two-Bottle Test

**Figure 11:**
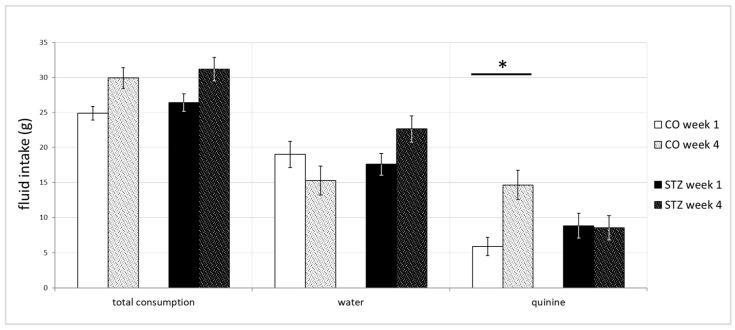
Fluid consumptions of the subacute deprivation session of the two-bottle test. Remarkable difference in the group fluid consumptions was detected in the subacute fluid deprivation session of the two-bottle test. The major deviation developed gradually, where the control group drank significantly more quinine solution after re-introducing the fluids during the fourth week, compared to their corresponding QHCl intake during the first week. (* denotes *p* ≤ 0.05).

Although the non-deprived fluid consumptions of the two groups practically did not differ at all, a significant difference was found in the three-hour consumptions in the fluid deprivation sessions of the experiment: the control group (n = 11) drank remarkably more quinine hydrochloride solution in the fourth week, compared to their QHCl consumption in the first week. Such differences were not present in the STZ-treated group (n = 12) as shown in Figure 11 (1st week, STZ, 8.83 ± 1.76 g, control, 5.89 ± 1.32 g; 4th week, STZ, 8.56 ± 1.74 g, control, 14.64 ± 2.5 g; Kruskal–Wallis, χ2(3) = 8.542, *p* < 0.05; control 1st week vs. control 4th week, Dunn, *p* < 0.05).

### 3.5. Taste Reactivity Test

**Figure 12:**
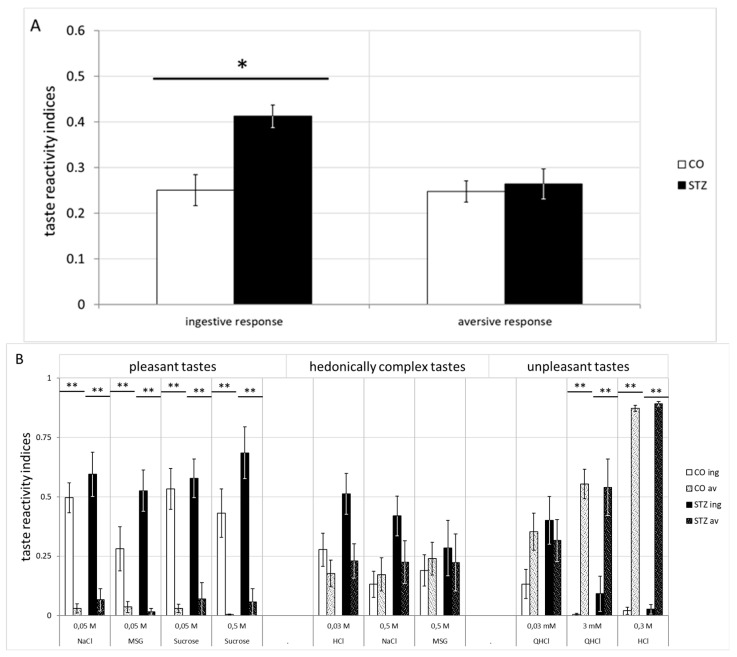

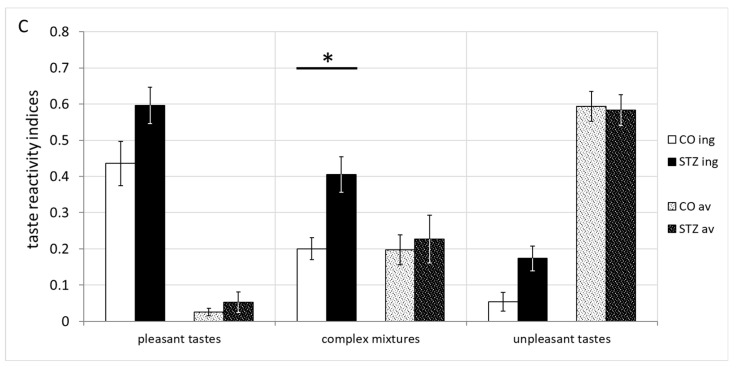
Differential taste reactivity indices of STZ-treated and control rats. (**A**) Indices of summed ingestive and aversive responses, (**B**) to the two concentrations of all taste stimuli, as well as (**C**) indices in case of grouped (pleasant, hedonically complex and unpleasant) gustatory stimuli. (* indicates *p* ≤ 0.05; ** represents *p* ≤ 0.001).

Significant preference score differences were observed between the control and the STZ-treated groups, with a clear ingestive dominance of the latter, in case of comparing the summed ingestive and aversive responses (Figure 12A) (χ2(3) = 12.765, *p* < 0.001; Dunn *p* < 0.05).

In case of the individual evaluation of the tastants (Figure 12B), a characteristic loss of significance was observed, mostly in the case of the hedonically complex tastes: in detail, the 0.03 M HCl, 0.05 M NaCl, 0.5 M MSG as complex; and 0.03 mM QHCl as unpleasant (0.05 M NaCl, χ2(3) = 28.944, *p* < 0.001; 0.05 M MSG, χ2(3) = 17.035, *p* < 0.001; 0.05 M sucrose, χ2(3) = 39.453, *p* < 0.001; 0.5 M sucrose, χ2(3) = 20.692, *p* < 0.001; 3 mM QHCl, χ2(3) = 26.352, *p* < 0.001; 0.3 M HCl, χ2(3) = 34.770, *p* < 0.001; Dunn *p* < 0.001).

Significant ingestive taste reactivity indices were observed (Figure 12C) in the hedonically complex taste stimulations (χ2(3) = 9.772, *p* < 0.001; Dunn *p* < 0.05).

## 4. Discussion

The present series of microelectrophysiological experiments elucidated the relatively high proportion of special chemosensory neurons that showed changes in firing rate in response to microosmoelectrophoretically administered D-glucose. Almost every fifth of the ACC neurons proved to be constituting elements of the forebrain GM neuronal network. The anterior cingulate cortical GM units represented both types of the chemosensory cells: ¾ of them were identified as being the excited GE-type neurons, whereas ¼ of them were identified as being the inhibited GI-type neurons. The ACC, partially based on observations of similar microelectrophysiological experiments in the rhesus monkey (yet unpublished data), is a long-predicted cortical site where both types of GM neurons have been found. In fact, it has joined the list of recently extensively investigated diencephalic structures, which includes the well-known hypothalamic nuclei, such as the LHA, VMH, arcuate, PVN, and DMH, as well as a group of inter-related forebrain regions that have been the focus of studies by our research group in the past decades: the globus pallidus [38,42], the nucleus accumbens [43], the mediodorsal prefrontal cortex [66], and the orbitofrontal cortex [44]. In the case of the former, excitingly rich, broadscale experimental findings have been reported about the differential, metabolism-dependent or independent glucose-sensing mechanisms of the GE- and the GI-type GM neurons [67]. As far as the latter is concerned, we have chosen a genuinely different experimental approach, implemented in both rat and monkey examinations, and based on our traditions (those of the Physiology Institute here, in Pécs), we have placed emphasis on studying the humoral, gustatory, homeostatic, as well as motivational–cognitive–emotional–behavioral aspects of the organism’s well-being. In these investigations, and this applies to the ACC as well, we could not compare the basic (elementary, e.g., molecular biological factors of glucose-sensing) attributes of the two types of GM neurons. Instead, different comparisons were made with the aim to unravel the differential specificity of the chemosensory cells in contrast to what characterizes the GIS neurons. These include a rather heavy set of functional parameters, such as the examination of specific neurochemical sensitivities, activity dependence or independence on motivation (role of hunger), distinct firing rate changes while conducting a high-fixed-ratio bar press feeding task, neuronal responsiveness to food or water deprivation, and other homeostatic challenges, and many simple and complex tests related to gustatory information processing have been performed between the group of the chemosensory neurons and that of the GIS units.

The glucose-monitoring neurons of the limbic forebrain, in general, are well known for their involvement in feeding–metabolic and motivational–behavioral control mechanisms [10,11,19,44,68,69], and this involvement also stands, more specifically, for the local chemosensory cells in the anterior cingulate cortex [46,70,71]. Our present and previous findings and the literature data substantiate that these limbic forebrain GM neurons are especially indispensable in the monitoring of relevant internal and external environmental signals, as well as integration and organization of adaptive mechanisms of homeostatic and behavioral control processes [28,29,30]. The present experiments have revealed that selective destruction and elimination of the GLUT2-equipped GM neurons in the ACC—of distinguished significance within the limbic forebrain neural circuitry [46,72,73,74]—lead to complex, adverse functional consequences: on the one hand, the intricate local morphological inter-relationships are disorganized or destroyed; on the other hand, utilization of the originally well-functioning action flexibility and sustained organismic control are also disturbed [75]. Taking the whole dataset together, there remains no doubt at all that loss of the local chemosensory neurons in the ACC elicits complex and multiple feeding-associated gustatory disturbances as well as characteristic behavioral physiological alterations.

Nevertheless, it is obligatory to note here that our present results must also be evaluated in a much broader context, from a much more distant perspective, with reference to the recent findings in non-human primates and human subjects. The primate brain, and especially this part of it, even that of the rhesus monkey’s, is much more developed and remarkably much more heavily wired, which provides it, in sharp contrast to the ‘grouped block type’ information processing in rodents, with much more flexibility in the organization and actualization of goal-directed operation of the above functional elements [26].

The literature data show us that the strongest afferentation to any cortical region in the brain is represented by the very dense dopaminergic fibers heavily innervating the anterior cingulate cortex [76,77]. In the light of the above observations, our present findings ((1) the dopamine-sensitive neurons represent almost 20% of all neurons tested in the ACC; (2) GM cells also represent about 20% of all ACC neurons tested; and (3) approximately 50% of all GM units here are dopamine-sensitive) may imply important considerations. On the one hand, it is reasonable to suppose now that the dopamine-sensitive GM cells form the decision-making capacity of a big portion of all ACC neurons. Consequently, on the other hand, this special group of the anterior cingulate cortical chemosensory units can represent a very appropriate target for new highly effective therapeutic approaches.

It is important to note here that, even recently, several studies have discussed the role of dopamine in regulatory processes of food and fluid intake behaviors and their implications for various feeding-related functional disturbances [78,79,80,81]. Acceptably, the damage of dopaminergic pathways in the ACC, in addition to also eliciting other broadly discussed functional deficits, causes impairment in the estimation of reward value of food and also in the desire to eat [78]. These alterations in the incentive motivation are usual findings of relevant lesion studies in this field.

The results of our preliminary microelectrophysiological recording along with taste stimulation experiments, especially those elucidating the 80% taste-responsiveness of GM units in the ACC, provide undisputable strong evidence for the intimate involvement of these chemosensory cells in the highest level of gustatory information processing.

The findings of our two-bottle and taste reactivity tests provided clear evidence for the intricate complexity of the gustatory information processing at the level of the cingulate cortex.

The results of these two series of taste-perception-associated examinations suggest the existence of a kind of taste-quality-recognition deficit in rats in the STZ group: i.e., these animals accumulated more accepting, ingestive types of responses even to unpleasant taste stimuli (the stronger salt, umami and acid, and both quinine) compared to much lower response rates of the control rats. In apparent contradiction with the above, an unexpected increase in the quinine consumption of the control animals was also observed at the 4th week of the 24 h fluid deprivation paradigm. Nevertheless, there were no significant differences in the QHCl consumption of the two (STZ-treated and control) groups at any time point of the no-deprivation paradigm. Consequently, this finding could not indicate an increased quinine preference in the control animals, as this elevation was not seen in non-deprived two-bottle test measurements. It is rather likely to be an extraordinary response to the urgent need for fluid in thirst, i.e., it seems that the power of the homeostatic drive has simply overrun that of the hedonic one. It is also known that rats, especially in the longer term, relatively easily grow accustomed to the taste of quinine [82], so if animals are very thirsty, they will probably have a more accepting attitude towards a bottle whose content is already known by them as a safe non-harmful substance, eventually with a particular bitter taste. Nevertheless, considering these above inter-related findings, it appears to be reasonable to suppose that, in the control rats, more sensitive finely tuned GM neuron-associated control mechanisms operate for evaluating the relative vital significance of the various quality signals of the available food or fluid items compared to those active in the anterior cingulate cortex of STZ-microinjected animals. Furthermore, our experimental findings also allow us to take into consideration that, at the same time, GM neurons (or their failure) in the ACC could potentially also alter the emotional processing of the given situation [74], and this might affect, among other things, the hedonistic evaluation of particular tastes. The hedonic value, as one of the most determinant attributes of taste in an individual’s personal experience, greatly influences, if not determines, his or her actual food consumption. As such, our present and previous results appear to be sharply relevant with respect to the organism’s body weight control as well.

Several brain areas, such as the amygdala, the insula, the orbitofrontal cortex, or the ventral striatum [44,83,84,85,86,87], have already been shown to be involved in the high-level regulation of hedonic evaluation of tastes in rats [88]. Similarly to the behavioral observations in the above regions, the damage to the local GM neurons here and the decline of their functional capability apparently caused a perception shift in the STZ-treated animals. Nevertheless, the results indicate a probably more complex condition than a simple shift in the gustatory perception. The distinct results of the deprivation sessions, namely, the significantly increased quinine consumption of control animals without the change in the summed (both quinine and water) fluid intake, indicate that the neurotoxin-treated animals experience the situation differently, i.e., its emotional valence is different for them [74,89]. In other words, the STZ-treated animals may perceive and experience a given unpleasant taste more pronouncedly, so they will continuously choose water over quinine, but the “more pronounced” would not necessarily mean a negative experience. It should be noted that, though the neurotoxin-treated group showed significantly more ingestive responses to the hedonically complex tastes (corresponding to the mixture of pleasant and unpleasant tastants [65]), they still evaluated quinine as being unpleasant overall. It well fits in this line of consideration that human studies in eating disorders and severe obesity reported dysfunction or atrophy in Brodmann 25 and 32 areas, which are homologues to the anterior part of the CC area in the rodent brain [90] and characterized by pronounced finickiness to quinine [88].

To emphasize the human clinical relevance of our above cognitive–emotional–behavioral findings, it is worth noting that bilateral anterior cingulotomy was a unique neurosurgical treatment used for patients with severe emotional imbalance, and it was also effectively used for the treatment of enormously severe cases of depression, unbearable and unstoppable pain, extreme cases of obsessive–compulsive disorder or anxiety hysteria [91,92,93]. Without causing learning disabilities, the lesions also had sedative effects, with feelings of pleasantness and contentment of mood, additionally with the absence or lessening of hostility, fear, any phobias, depression, and obsessive thinking with reconstructing ego strength [89,94].

Despite all valuable ideas from previous considerations, it is important to note that alterations in even elementary mechanisms of glucose-sensing processes can interfere with the feeding behavioral output of the animal. Alterations in glucose-sensing may result in a condition which elicits increased food and fluid intake behavior. Deficit of food reward can also lead to hypophagic and/or hypodipsic behavior.

It should be noted that both single (20 min) and repeated anodal transcranial direct current stimulations have been shown to lower blood glucose levels, improve glucose tolerance, and reduce stress axis activity in human male volunteers, without affecting blood serum insulin concentrations. These findings may be of clinical interest for the treatment of hyperglycemia [95].

Some studies suggest that the condition of individuals suffering from diseases such as Alzheimer’s and similar neurodegenerative disorders can be improved by boosting brain glucose metabolism, namely by stimulating cerebral glucose uptake [96].

In addition, the increasingly widespread use of stem cell studies suggests the possibility that hypothalamic—one of the most important areas of glucose-monitoring neurons—neural stem cell-based therapy could be developed to control weight gain and glucose intolerance [97].

Our behavioral experiments ended with similar results to those of the abovementioned clinical bilateral anterior cingulotomy, i.e., lack of fear and anxiety in the open-field and elevated plus maze tests, as well as maintaining the memory integrity and physiological learning capability in the elevated plus maze test. We would like to emphasize here that results of our recently started pilot experiments on conditioned taste aversion also reinforced the well-working, maintained learning capability of the animals with STZ microinjection into the ACC (yet unpublished data). According to Brent A. Vogt, it should also be taken into consideration that the rat ACC reacts differently than that of primate species in the case of effort-related decision making: organismic responses are initiated with or without dopamine-related response elements. Nevertheless, one should also keep in mind the extraordinary significance of the primate ACC in dopamine-associated incentive motivation [98].

Based on all these findings, it appears reasonable to suppose that the local GM neurons in the anterior cingulate cortex are indeed intimately involved in the organization of adaptive control processes of individuals’ food and fluid intake behavior.

## 5. Conclusions

The present study was designed to elucidate multiple functional attributes of GM neurons of the ACC in feeding-associated control mechanisms. In the microelectrophysiological experiments, a relatively high proportion of GM neurons were identified here, in addition to recording a similar proportion of dopamine-sensitive neural cells among the ACC units. Even more excitingly, approximately half of all GM neurons proved to be dopamine-sensitive, whereas about 80% of the chemosensory units were responsive to gustatory stimulation. The loss of these GLUT2-containing chemosensory neural cells here led to the development of complex disturbances in taste information processing and organization of motivationally determined adaptive behavioral actions. Such deficits altogether seriously interfere with the flexible responsiveness of the individual to various differential, either internal or external, environmental challenges; thus, it is reasonable to suppose that these neurons are involved in central regulatory processes contributing to the maintenance of emotional–cognitive–homeostatic balance for the well-being of the organism.

Overall, we are convinced that the better understanding of fine details of the exact neural background of these complex regulatory disturbances will open new research lines in the field. Further on, hopefully, it will also support the invention of new highly effective therapeutic strategies and practices in feeding–metabolic disorders.

## Figures and Tables

**Table 1:** Microelectrophoretically applied dopamine responsiveness of GM and GIS neurons in the rat ACC.

	Dopamine		
	±	ø	Total
GM	7	8	15
GIS	10	67	77
Total	17	75	92
±: excitatory or inhibitory response			
ø: no response			χ(1) = 9.454, *p* < 0.05

## Data Availability

The datasets used and/or analyzed during the current study are available from the corresponding author on reasonable request.

## List of Abbreviations

Some of the frequently used symbols and abbreviations.

**Symbol/Abbreviation**	**Referred to**
*	statistical significance level of differences at *p* ≤ 0.05
**	statistical significance level of differences at *p* ≤ 0.001
ACC	anterior cingulate cortex
CC or cctx	cingulate cortex
CO	control
DA	dopamine
glut	glutamate
GLUT2	type 2 glucose transporter protein
GM	glucose-monitoring
QHCl	quinine hydrochloride
STZ	streptozotocin
